# VecTest as Diagnostic and Surveillance Tool for West Nile Virus in Dead Birds

**DOI:** 10.3201/eid1012.040836

**Published:** 2004-12

**Authors:** Ward B. Stone, Joseph C. Okoniewski, Joseph E. Therrien, Laura D. Kramer, Elizabeth B. Kauffman, Millicent Eidson

**Affiliations:** * New York State Department of Environmental Conservation, Albany, New York, USA;; † New York State Department of Health, Albany, New York, USA

**Keywords:** West Nile virus, birds, diagnosis, false positive reaction, RT-PCR, research

## Abstract

The VecTest antigen-capture assay for West Nile virus was performed on oral and tissue swabs from dead birds in New York State from April 2003 through July 2004. Results were compared with those from real-time reverse transcriptase–polymerase chain reaction of kidney or brain. Oral VecTest sensitivity is adequate for surveillance in American Crows (*Corvus brachyrhynchos*) (87%), Blue Jays (*Cyanocitta cristata*) (80%), and House Sparrows (*Passer domesticus*) (76%). Oral VecTest performed well for small samples of American Kestrels (*Falco sparverius*), Northern Cardinals (*Cardinalis cardinalis*), Common Grackles (*Quiscalus quiscula*), and House Finches (*Carpodacus mexicanus*). Poor sensitivity occurred in most raptors, Mourning Doves (*Zenaida macroura*), Fish Crows (*Corvus ossifragus*), and American Robins (*Turdus migratorius*). Specificity was excellent (98%), except for false-positive results that occurred mostly in Gray Catbirds (*Dumatella carolinensis*), Green Herons (*Butorides virescens*), and tests of blood and tissues. Feather pulp and kidney may be useful for VecTest assays in corvids.

## Abstract 2

The VecTest WNV assay is adequate for diagnostic and surveillance purposes in American Crows, Blue Jays, and House Sparrows.

After West Nile virus (WNV) was discovered in birds, horses, and humans in New York State in 1999 (*1*), the New York State Department of Health established a surveillance system to follow seasonal and geographic trends in WNV activity (*2*). An important part of this system is testing dead birds for WNV. Detection in bird species is used as an early warning system to measure the threat of the virus to humans and as a threshold indicator for mosquito control programs. Surveillance is also used to assess the impact of WNV on avian populations and to document geographic, seasonal, and annual trends.

In the dead bird testing program, birds are reported by the public and submitted (largely through county health departments) to the Wildlife Pathology Unit of the New York State Department of Environmental Conservation. The Wildlife Pathology Unit necropsies priority birds and collects tissues, which are then sent to the health department's Arbovirus Laboratory, where they are tested for WNV RNA by real-time reverse transcriptase–polymerase chain reaction (RT-PCR) (*3*). Since WNV was detected in New York in 1999, the Wildlife Pathology Unit and the Arbovirus Laboratory have processed >19,000 specimens as part of the surveillance program. The elapsed time between the initial reporting of a dead bird and posting of the RT-PCR results on the surveillance system's Health Information Network, an Internet-based data and information tracking system (*4*), can be as long as 3 weeks. Along with faster processing of specimens, a simpler alternative to the RT-PCR test was desired to quicken the actual WNV testing and reporting and to reduce personnel time and expense.

In 2000, a rapid antigen-capture wicking assay in a dipstick format (VecTest, Medical Analysis Systems, Camarillo, CA) was introduced for detecting WNV in mosquito pools (*5**,**6*). The VecTest requires a minimal amount of supplies and equipment and is easy to use; its results are available 15 minutes after the dipstick is placed in the sample solution. After high WNV titers were demonstrated in the oral and cloacal cavities of corvids (*7*), the VecTest was evaluated relative to RT-PCR in saliva, feces, and tissue samples from a small sample of American Crows (*Corvus brachyrhynchos*) in Illinois (*8*), and oral and cloacal swabs from corvids in a larger study in Canada (*9*). The latter study reported a sensitivity of approximately 83% for American Crows.

The objective of this study was to determine whether the VecTest antigen assay would be useful for WNV surveillance in New York State. We compared the results of oral VecTests with RT-PCR of tissue for a large number of birds of various species. In addition, we explored use of the VecTest with swabs from the cloaca, feather pulp, and internal tissues to determine whether use of other antigen sources might improve the sensitivity of the test.

## Methods

Birds included in this study were those found dead in New York State, from April 2003 to July 2004, and received at the Wildlife Pathology Unit for WNV testing from county health departments, veterinarians, wildlife rehabilitators, other organizations and agencies, and the general public. Some specimens were frozen before delivery to the Wildlife Pathology Unit. Although locally submitted birds were often examined and necropsied on the day of receipt, most specimens were not processed until the following day, or later. Carcasses were held at 4°C until necropsied. The selection of specimens for testing was not usually related to postmortem condition. The highest priority was placed on corvids, raptors, and House Sparrows (*Passer domesticus*), while low priority was given to Rock Doves (*Columba livia*), European Starlings (*Sternus vulgaris*), and Common Grackles (*Quiscalus quiscula*). At times, high priority was placed on birds from specific geographic areas for which local health departments had requested immediate testing.

At necropsy, oral swab samples were collected with sterile, polyester fiber-tipped plastic applicators by moving the tip vigorously around the entire oropharyngeal cavity and, by July 2003, the proximal esophagus. The swab was then twirled for 3 to 5 s in 1.0 mL of the VecTest buffer solution (provided with the VecTest kit) in 10-mL plastic tubes, pressing the tip against the side of the tube. The swab was then discarded.

For a number of different bird species, swabs were taken from kidney, liver, heart blood, cloaca, brain, and feather pulp, in addition to the oropharyngeal cavity. Swabs from kidney and liver were obtained by sticking the applicator tip into the parenchyma and rotating the tip to ensure capture of tissue. Heart blood swabs were obtained by immersing the tip in blood contained within the atria or right ventricle. Cloacal swabs were obtained by moving the tip vigorously against the mucosal lining. Brain swabs were taken by running the tip through cerebral gray and white matter. Feather pulp was taken by pulling a blood feather from the wing or tail and then expressing the pulp onto a swab. All swab samples were then mixed into separate VecTest buffer solutions as described above.

In a class II biosafety cabinet at the Wildlife Pathology Unit, 0.25 mL of each swab solution was transferred to a conical microfuge tube (supplied with the kit), the VecTest strip was inserted into the tube, and results were read in the fluorescent light of the safety cabinet 15–30 min later. According to manufacturer's instructions, the development of a reddish purple line, in both the test and control zones of the VecTest strip, was recorded as a positive result. Any test strip that did not develop a control line was discarded; then, 0.25 mL of the original solution was pipetted into another microfuge tube, and a new strip was used to test the solution. Observations on the intensity of positive results, as well as descriptions of any unusual features, were also recorded.

Swab samples in VecTest buffer solution that were not tested on the day of collection were refrigerated overnight at 4°C. If a delay of >24 h occurred before testing, solutions were frozen at –20°C. To determine the effects of refrigeration on the buffer solution, a limited number of swab samples were held at 4°C for intervals of 3 to 7 days. A similar study was conducted by freezing swab solutions at –20°C for 2 days to 7 months to determine if freezing would affect VecTest results. Most VecTest strips used throughout the study contained a single antigen-binding site specific for WNV; however, 500 test strips used during June and July 2003, and in July 2004, included additional test zones for eastern equine encephalitis and St. Louis encephalitis.

Tissues for RT-PCR testing were obtained during necropsy with single-use sterile disposable scalpels and stainless steel forceps and scissors. After use, forceps and scissors were placed in Promicidal disinfectant (Chemsearch Industries, Irving, TX) for later cleaning and steam autoclaving at 120°C for 20 min at 1.0 kg/cm^2^. Harvested tissues were immediately placed in individual 1-oz plastic jars and capped. Jar lids were labeled with the individual specimen number, species name, and tissue type, and the jar sides with the specimen number. The plastic jars containing the tissues were then frozen at –80°C before transport to the state Arbovirus Laboratory for RT-PCR testing.

At the Arbovirus Laboratory, RNA was extracted from kidney or brain tissue by ABI Prism 6700 robotic workstation (Applied Biosystems, Foster City, CA) or RNeasy (Qiagen, Inc., Valenci, CA) and assayed for WNV by real-time RT-PCR using ABI Prism 7700 or 7000 sequence detectors, as described previously (*3**,**10*). Briefly, each sample was tested with two sets of primer probes, targeting the envelope or NS1 region of the WNV RNA. Controls consisted of a set of WNV RNA standards that ranged from 0.08 to 90 PFU per sample, and WNV-positive bird tissue that was prepared and RNA-extracted with the assay. The sensitivity of the real-time RT-PCR assay is 0.08 PFU or 40 copies of RNA. A sample was declared positive only if WNV was detected with both primer-probe sets. Differences in VecTest performance in data subsets of interest were assessed by chi-square analysis. Data are expressed as a percentage in the text and tables only where n > 10.

## Results

Results from VecTests of oral swabs and RT-PCR of kidney or brain from 2,913 birds (116 species, 16 orders) were compared (Table 1); of these, 1,013 (35%) were positive for WNV by RT-PCR. The sensitivity of the oral VecTest in RT-PCR–positive birds was 87% in American Crows, 80% in Blue Jays (*Cyanocitta cristata*), and 76% in House Sparrows. WNV was detected by RT-PCR in small numbers (n = 1–16) of 29 additional species, and confirmed by oral VecTest in 11 of these species. In those 11 species, despite small sample sizes, results suggested some species-specific variability in sensitivity. The test detected WNV in >50% of RT-PCR–positive birds of the following species: American Kestrels (*Falco sparverius*) (3/4), Northern Cardinals (*Cardinalis cardinalis*) (4/6), Common Grackles (3/6), and House Finches (*Carpodacus mexicanus*) (7/7) but was unable to detect WNV in RT-PCR–positive Red-tailed Hawks (*Buteo jamaicensis*) (0/10) and Great Horned Owls (*Bubo virginianus*) (0/12). Poor VecTest sensitivity was also recorded in small numbers of Mourning Doves (*Zenaida macroura*) (0/6), American Robins (*Turdus migratorius*) (3/16), and Fish Crows (*Corvus ossifragus*) (2/10).

**Table 1:** Comparison of oral VecTest and RT-PCR results^a,b^ for West Nile virus in dead birds, New York State, April 2003– July 2004

Species (listed by order)	No. of birds			
	No. birds tested	RT-PCR–positive	VecTest results^c^	
			True positive (%)^d^	False-positive (%)^e^
Ciconiiformes				
Great Blue Heron (*Ardea herodias*)	8	1	0	0
Anseriformes				
Mallard (*Anas platyrhynchos*)	7	1	0	0
Falconiformes				
Bald Eagle (*Haliaeetus leucocephalus*)	6	1	0	0
Sharp-shinned Hawk (*Accipiter striatus*)	14	3	1	0
Cooper's Hawk (*Accipiter cooperii*)	33	3	0	0
Northern Goshawk (*Accipiter gentilis*)	3	1	0	0
Broad-winged Hawk (*Buteo platypterus*)	1	1	1	NA
Red-tailed Hawk (*Buteo jamaicensis*)	36	11	0 (0)	0
American Kestrel (*Falco sparverius*)	12	4	3	0
Merlin (*Falco columbarius*)	4	1	0	0
Peregrine Falcon (*Falco peregrinus*)	7	1	0	0
Galliformes				
Impeyan Pheasant (*Lophophorus impeyanus*)	1	1	0	0
Charadriiformes				
Herring Gull (*Larus argentatus*)	3	1	0	0
Great Black-backed Gull (*Larus marinus*)	3	2	1	0
Columbiformes				
Mourning Dove (*Zenaida macroura*)	75	6	0	0
Strigiformes				
Great Horned Owl (*Bubo virginianus*)	25	12	0 (0)	1
Passeriformes				
Blue Jay (*Cyanocitta cristata*)	339	166	133 (80)	1 (1)
American Crow (*Corvus brachyrhynchos*)	1,076	702	608 (87)	7 (2)
Fish Crow (*Corvus ossifragus*)	22	10	2 (20)	0
Common Raven (*Corvus corax*)	2	1	0	0
American Robin (*Turdus migratorius*)	233	16	3 (19)	1 (1)
Gray Catbird (*Dumatella carolinensis*)	113	2	0	13 (12)
Brown Thrasher (*Toxostoma rufum*)	2	1	0	0
European Starling (*Sturnus vulgaris*)	85	1	1	1 (1)
Scarlet Tanager (*Piranga olivacea*)	5	1	0	0
Northern Cardinal (*Cardinalis cardinalis*)	21	6	4	0
Common Grackle (*Quiscalus quiscula*)	189	6	3	1 (1)
Brown-headed Cowbird (*Molothrus ater*)	8	1	0	1
House Finch (*Carpodacus mexicanus*)	17	7	7	1
House Sparrow (*Passer domesticus*)	222	41	31 (76)	1 (1)
Gouldian Finch (*Chloebia gouldiae*)	1	1	0	0
Society Finch (*Lonchura domestica*)	1	1	1	NA
Other species^f^	339	0	NA	8^g^ (2)
Total all species	2,913	1,013	799 (79)	36 (2)

^a^RT-PCR, reverse transcriptase-polymerase chain reaction; NA, not available. ^b^Principally tests of kidney (brain or heart alternate when kidney missing). ^c^Percentages are provided for species where n > 10. ^d^No. and percentage of RT-PCR–positive birds that were VecTest-positive. ^e^No. and percentage of RT-PCR–negative birds that were VecTest-positive. ^f^84 species representing 16 orders. ^g^Mute Swan (*Cygnus olor*) (1), Green Heron (*Butorides virescens*) (6), Northern Mockingbird (*Mimus polyglottos*) (1).

VecTest sensitivity did not appear to be seriously compromised by extensive postmortem deterioration or freezing of the carcasses. RT-PCR–positive American Crows (n = 124) and Blue Jays (n = 30) that showed moderate or more severe autolysis of tissues, including extensive maggot activity in many, were positive by VecTest in 89% and 87% of cases, respectively. VecTest sensitivity in birds that had been frozen was 80% (49/61) in American Crows and 79% (49/62) in Blue Jays. In addition, the freezing (–20°C) of 18 VecTest-positive oral swab sample solutions for 2 days to 7 months had no effect on results. All repeat tests were positive at what appeared to be the same intensity.

Nineteen oral swab samples in buffer solution from American Crows and Blue Jays were tested with VecTest strips, and then refrigerated at 4°C for 3 to 7 days. Samples were then retested with VecTest strips, and all pre-, and postrefrigeration results were the same. Seventeen of the samples tested were positive, and 2 were negative in both phases of testing.

False-positive results in oral VecTests were observed in 36 (2%) of 1,900 RT-PCR–negative birds and rarely occurred in species, with the exception of Gray Catbirds (*Dumatella carolinensis*) (12%, 13/111) and Green Herons (*Butorides virescens*) (75%, 6/8) (Table 1). Thus, the overall specificity (identifying an RT-PCR-negative as negative) of the VecTest was high (98%, 1,864/1,900), as were the VecTest–positive predictive value (96%, of 835 VecTest-positive birds, 799 were also RT-PCR-positive) and –negative predictive value (90%, of 2,078 VecTest-negative birds, 1,864 were also RT-PCR-negative).

Most (24/36) of the false-positive results, including all those involving Gray Catbirds and Green Herons, consisted of very narrow lines at the lower border of the test region, unlike the full-width colored bands described in the manufacturer's instructions as positive results, and recorded in oral tests of RT-PCR–positive birds (Figure). These lines, in contrast with the VecTest-positive results, which usually developed to their full extent within 10 min, often continued to intensify beyond 15 min (sometimes only noticeable after >15 min had elapsed). Narrow-line results were not identified in oral tests of RT-PCR–positive birds, but such results could have been merged with true-positive wide-band results in tests where wide-band results developed. At least four of the other false-positive results were faint positive reactions in multiple test zones of the WNV/St. Louis encephalitis/eastern equine encephalitis version of the VecTest; two of the false-positive results appeared intermediate, between narrow-line and wide-band results. The other six false-positives also were obtained with the WNV/St. Louis encephalitis/eastern equine encephalitis test. No distinct wide-band–positive results were obtained in RT-PCR–negative birds.

**Figure:**
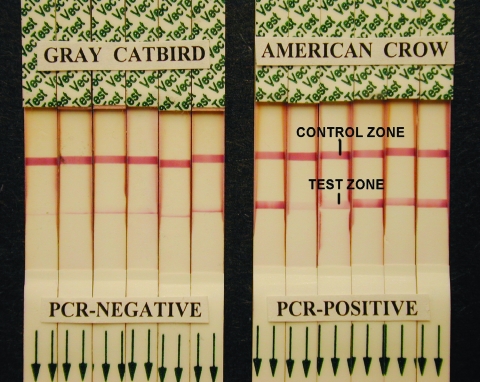
West Nile virus (WNV) VecTest results from oral swabs of Gray Catbirds showing narrow-line false-positive results compared with typical true-positive VecTest results from reverse transcriptase–polymerase chain reaction–positive American Crows. Note the near exclusive deposition of pigment at the lower margin of the test zone on the dipsticks of catbirds, and the distribution of pigment across the full width of the test zone in the WNV-positive crows, even in very weak positive tests.

VecTest results for swabs taken from the cloaca, heart blood, and kidney of RT-PCR–positive corvids and House Sparrows produced results generally similar to those obtained from oral swabs (Table 2). When oral tests were positive (n = 62), tests of the other three tissues were almost uniformly positive (cloacal [95%], heart blood [97%], kidney [98%]). When oral tests were negative (n = 22) in these RT-PCR–positive birds, however, positive VecTest results (often weak) were sometimes recorded in cloacal (27%), blood (14%), and kidney (55%) samples. VecTest of oral swabs, and swabs of alternate tissues, for 18 RT-PCR–positive raptors (Table 2) were negative, with the exception of one American Kestrel (oral) and one Great Horned Owl (kidney). Narrow-line results recorded in three heart blood samples and one kidney sample from these raptors were not included as positives in Table 2.

**Table 2:** Comparative sensitivity of VecTest with swabs from different sources in RT-PCR–positive birds^a,b^

Species	N	No. positive (% positive^c^) by VecTest			
		Oral	Cloacal	Heart blood	Kidney
Blue Jay	37	29 (78)	31 (84)	28 (76)	33 (89)
American Crow	36	24 (67)	25 (69)	25 (69)	30 (83)
House Sparrow	11	9 (82)	9 (82)	10 (91)	10 (91)
Raptors^d^	18^e^	1 (6)^f^	0	0	1 (6)^g^

^a^RT-PCR, reverse transcriptase–polymerase chain reaction. ^b^Four sources from each bird, with exceptions noted in footnote e. ^c^Wide band positive only (see text). ^d^Sharp-shinned Hawk (1), Cooper's Hawk (1), Red-tailed Hawk (7), American Kestrel (1), Merlin (1), Peregrine Falcon (1), Great Horned Owl (6). ^e^n = 14 for cloaca; n = 15 for heart blood. ^f^Positive VecTest in one American Kestrel. ^g^Positive VecTest in one Great Horned Owl.

In RT-PCR–negative birds, VecTests of internal tissues (Table 3) produced narrow-line false-positive results (as described above and in the Figure) far more frequently than occurred in tests of oral swabs. These lines were most common in heart blood (30%, 62/208 RT-PCR-negative birds). As with their occurrence in oral testing, these lines were also far more prevalent in Gray Catbirds. Tests of cloacal swabs produced results similar to the oral testing (4% false-positives, 5/117; all were in catbirds).

**Table 3:** Frequency of false-positive VecTest results^a^ in tests of swabs from cloacal and tissue sources in RT-PCR–negative birds

Species	No. false-positive/no. RT-PCR–negative (%)			
	Cloacal	Kidney	Liver	Blood
Cooper's Hawk	0/3	1/7	1/2	1/9
Mourning Dove	0/2	0/7	0/4	0/7
Blue Jay	0/3	3/26 (12)	0/8	0/8
American Crow	0/23 (0)	19/112 (17)	6/51 (12)	14/47 (30)
American Robin	0/9	3/30 (10)	0/13	4/14
Gray Catbird	5/15 (33)	16/28 (57)	4/15	16/18
European Starling	0/8	4/30 (13)	3/11	3/5
Common Grackle	0/9	0/55 (0)	1/25 (4)	4/12
House Sparrow	0/9	0/15	1/5	4/15
Other avian	0/36 (0)	9/87 (10)	1/29 (3)	16/73 (22)
All species	5/117 (4)	55/397 (14)	17/163 (10)	62/208 (30)

^a^Principally narrow lines at the lower margin of the test zone on the dipstick; RT-PCR, reverse transcriptase–polymerase chain reaction.

VecTest of brain tissue in 49 oral-negative, RT-PCR–positive birds yielded eight wide-band–positive results (Table 4). Sensitivity in corvids (21%, 3/14) was similar to that in raptors (18%, 5/28). Five narrow-line results were also recorded in raptors (two Sharp-shinned Hawks (*Accipiter striatus*), three Red-tailed Hawks). VecTest results of brains from 17 RT-PCR–negative raptors (including eight Red-tailed Hawks) were negative.

**Table 4:** VecTest results from brain swabs of RT-PCR–positive^a^ birds with oral VecTest-negative results

Species	N	No. positive	No. narrow-line results^b^
Sharp-shinned Hawk	2	0	2
Cooper's Hawk	2	0	0
Northern Goshawk	1	1	0
Red-tailed Hawk	11	4	3
American Kestrel	1	0	0
Peregrine Falcon	1	0	0
Great Horned Owl	10	0	0
Blue Jay	4	0	0
American Crow	9	3	0
Common Raven	1	0	0
Other species^c^	7	0	0
All species	49	8	5

^a^RT-PCR, reverse transcriptase–polymerase chain reaction. ^b^Type of result most commonly seen in internal tissues of RT-PCR–negative birds (see text). ^c^Great Blue Heron (1), Mallard (1), Herring Gull (1), Great Black-backed Gull (1), Mourning Dove (2), Scarlet Tanager (1).

VecTest of feather pulp for 43 RT-PCR–positive corvids (37 American Crows, 5 Blue Jays, 1 Fish Crow) identified WNV in 36 (84%) of the birds tested. VecTest of oral swabs identified WNV in 32 (74%) of the same birds. VecTest specificity for feather pulp was 99%; the test correctly identified 93 of 94 RT-PCR–negative corvids as negative.

Oral VecTest sensitivity in American Crows early in the WNV season (April – June) was poor 17% (1/6) in 2003 but was 82% (14/17) during the same period in 2004. No difference in sensitivity was found in tests of hatch year (86%, 162/188) and after hatch year (87%, 418/480) American Crows. Sensitivity of the oral VecTest in Blue Jays was somewhat higher in after hatch year birds (86%, 57/66) than hatch year birds (75%, 71/95), but the difference was not significant (p < 0.10).

The VecTest analyses showed low levels (2.6%; 26/1,013) of false-negatives in RT-PCR testing. Twenty-six birds with positive, broad-lined, VecTest results and initially negative RT-PCR results were positive on subsequent tests, which included RT-PCR of original or reextracted sample, indirect fluorescent-antibody assay of cell culture–isolated virus, or assay of an alternate tissue. This group consisted of 15 American Crows, 6 Blue Jays, 2 House Finches, 2 Northern Cardinals, and 1 House Sparrow.

## Discussion and Conclusion

The sensitivity of oral VecTest reported here for American Crows and Blue Jays in New York State was similar to that reported in smaller scale evaluations (*8**,**9*) and appears acceptable for seasonal and geographic surveillance, provided an adequate supply of these corvids exists for testing. Our study further suggests that oral tests of House Sparrows, House Finches, and Northern Cardinals, three common urban or suburban species, might be efficiently used to survey for WNV in some areas where corvid populations have been diminished by WNV (*11*) or are uncommon for other reasons.

Komar et al. (*7*) detected high WNV titers in cloacal and oral swabs from experimentally infected corvids, a finding that indicates that both of these orifices may be useful for virus detection. In our comparison, the sensitivity of the VecTest for detecting WNV in both cloacal and oral swabs from American Crows was similar (69% and 67%, respectively). Lindsay et al. (*9*), however, found that VecTests of cloacal swabs were less sensitive than oral VecTests for detecting WNV in American Crows (58.3% and 92.8%, respectively). This discrepancy may be due to limited sample sizes in both studies and warrants further comparison of the two swabs. Lindsay et al. (*9*) demonstrated that swab solutions could be held up to 7 days at temperatures ranging from –20°C to 18°C. Our data also showed that neither freezing of the swab samples, at –20°C for 2 days to 7 months, nor refrigeration at 4°C for 3 to 7 days, had any effect on the sensitivity of the VecTest.

When testing will include or be limited to oral sampling, we suggest the following protocol to help standardize the technique and maximize the amount of tissue and fluid captured by the swab tip. After moving the swab tip against the lining of the mouth, compress the throat immediately behind the head and vigorously move the swab tip within the constricted entrance to the esophagus. This aggressive technique should be used only in dead birds.

Recent findings have shown vascular flight feather pulp of corvids to be a superior source for WNV isolation by culture (*12*). Our limited data showed feather pulp to be slightly more sensitive than oral swabs for detecting WNV with VecTest assays in corvids, and feather pulp specificity was excellent. Using feather pulp as an antigen source for VecTest assays may be advantageous, especially for testing live birds or where oral samples from dead birds may be compromised by autolysis or contamination. However, whether feather pulp or some other tissue would be useful in detecting WNV in species for which oral swabs appear ineffective requires further evaluation. The results obtained with other tissues from Great Horned Owls and Red-tailed Hawks were not encouraging. In experimentally exposed corvids, WNV has been shown to be present at roughly similar concentrations in a wide variety of tissues at death (*13*). A similar study of viral distribution and concentration in species, such as raptors and songbirds like robins, that showed poor VecTest results would be useful. A substantial fraction of birds from this group would likely have died from other causes (e.g., traumatic injury, poisoning) during periods when antigen levels were low. Low antigen levels and poor VecTest sensitivity may occur in WNV-susceptible species early in the incubation period or during recovery.

In this study, VecTest of oral swabs correctly identified RT-PCR–negative birds as negative in most cases (high specificity), which was consistent with results obtained in a similar study (*9*). Yaremych et al. (*8*), however, reported lower specificity with Illinois birds but, as mentioned by the authors, this finding may have been due to small sample size in their analysis. Also, the Illinois study tested a mixture of fecal, saliva, and tissue samples, which may not be directly comparable to tests of oral swabs alone. Lower specificity occurred in our study in oral tests of Gray Catbirds and Green Herons and in tests of internal tissues in a variety of species. VecTest results in these tests all involved the occurrence of the narrow-line false-positives mentioned earlier. The cause of these potentially misleading lines in the WNV test region of the dipstick was not determined. Although they can be readily distinguished from true-positive results in most cases, their elimination from this assay should be a high priority for the manufacturer of the VecTest. The number of species showing narrow-line results will likely increase as this test is used on a wider array of avian species. Also, in rare cases, results appear equivocal even to experienced test evaluators. In the interim, we recommend that the VecTest instruction sheet be modified to alert users to this phenomenon.

The VecTest has many attributes that make it a useful substitute for RT-PCR or other more complicated techniques. It is fast, easy to use, relatively inexpensive, and can be readily employed in the field. The VecTest has good sensitivity in key WNV–vulnerable species, can potentially be used with a variety of tissue sources, and has similar efficacy in fresh and decomposed carcasses. Clear wide-band–positive results have to date shown a 100% positive predictive value. The most serious disadvantages of the VecTest are its poor sensitivity in some species, and the narrow-line false-positive results. In situations in which improved sensitivity is desired, testing of kidney could reduce the number of false-negatives in American Crows, Blue Jays, and House Sparrows. However, the use of internal tissues requires opening the body cavity and increases human risk for WNV exposure. We recommend RT-PCR or other backup for negative results in cases where detection is critical, for diagnostic work, and in assessing threats to humans or animals.

In addition to its use in surveillance activities, the VecTest could be used as a diagnostic tool in some veterinary practices (e.g., zoos, exotic birds), some wildlife rehabilitation operations, and by biologists studying illness and death in wildlife. Both surveillance and diagnostic applications would benefit greatly from new findings concerning the test's sensitivity relative to a much larger array of avian species. Similar evaluation of the VecTest relative to amphibians, reptiles, and mammals also would be useful.
